# Second‐line MRI after expert transvaginal ultrasound in selected patients with suspected complex deep endometriosis: A prospective cohort study

**DOI:** 10.1111/aogs.70372

**Published:** 2026-10-23

**Authors:** Mira Rehal, Malin Brunes, Chikako Suzuki, Elisabeth Epstein

**Affiliations:** ^1^ Department of Clinical Science and Education Karolinska Institutet, South General Hospital Stockholm Sweden; ^2^ Department of Obstetrics and Gynecology South General Hospital Stockholm Sweden; ^3^ Department of Diagnostic Radiology Stockholm South General Hospital Stockholm Sweden; ^4^ Department of Molecular Medicine and Surgery Karolinska Institutet Stockholm Sweden

**Keywords:** diagnostic imaging, endometriosis, magnetic resonance imaging, surgical planning, transvaginal ultrasound

## Abstract

**Introduction:**

The objective of this study was to evaluate the added diagnostic yield of second‐line magnetic resonance imaging (MRI) after expert transvaginal ultrasound (TVS) for preoperative mapping of deep endometriosis (DE).

**Material and Methods:**

In this prospective observational cohort study, women with suspected DE scheduled for surgery after expert ultrasound were included. TVS was performed according to the International Deep Endometriosis Analysis (IDEA) protocol. MRI was selectively performed as a second‐line investigation in patients with suspected advanced disease, inconclusive TVS, or advanced symptoms despite negative TVS findings. Surgical visualization served as the primary reference standard, with histopathology used as a secondary reference standard when available. Diagnostic performance (sensitivity, specificity, predictive values, likelihood ratios, and Cohen's *κ*) was assessed for TVS, MRI, and their combination, and stratified by prior surgical status. For the combined approach, a positive result was defined as a positive finding on either modality (TVS or MRI), representing a rule‐out strategy.

**Results:**

Among 221 women with suspected deep endometriosis, 189 underwent surgery, of whom 112 had both TVS and MRI; 55 had previous abdominal surgery. Combined imaging improved sensitivity for rectosigmoid DE (+10.3%; +6 true positives (TP), +3 false positives (FP)) and uterosacral ligament involvement (+29.2%; +14 TP, +12 FP) but reduced specificity for endometriomas (−19.0%; true negatives (TN) 18 to 14), obliteration of the pouch of Douglas (−13.3%; TN 14 to 8), and uterosacral ligaments (−20.3%; TN 35 to 23). Most lesions missed by TVS were in the mid or upper rectum. Accuracy for the urinary bladder and ureters decreased with the addition of MRI (82.2% to 75.7%). Sensitivity for rectosigmoid lesions was higher with the combined method (TVS + MRI) in women with prior surgery.

**Conclusions:**

Among selected complex patients undergoing second‐line MRI after expert TVS, combined imaging increased detection of rectosigmoid and uterosacral ligament lesions, but at the cost of reduced specificity. The sensitivity for rectosigmoid disease was greatest among patients with prior abdominal surgery, although this subgroup finding should be considered exploratory. These findings are consistent with a selective rather than routine role for MRI after expert TVS.

## Graphical abstract

Second‐line MRI improves detection beyond expert transvaginal ultrasound in patients with suspected complex deep endometriosis, particularly for bowel and uterosacral ligament lesions, but at the cost of reduced specificity. These findings are consistent with a selective rather than routine role for MRI after expert transvaginal ultrasound.

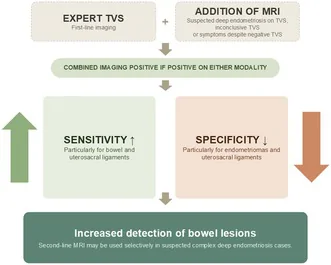

**Abbreviations**
BMI: body mass index
CI: confidence interval
DE: deep endometriosis
IDEA: International Deep Endometriosis Analysis
MRI: magnetic resonance imaging
NPV: negative predictive values
PPV: positive predictive values
TVS: transvaginal ultrasound

**Key message**

Second‐line MRI improves detection beyond expert transvaginal ultrasound in patients with suspected complex deep endometriosis, particularly for bowel and uterosacral ligament lesions, but at the cost of reduced specificity. These findings are consistent with a selective rather than routine role for MRI after expert TVS.

## 1 INTRODUCTION

Surgical excision of deep endometriosis (DE) presents considerable challenges due to adhesions and fibrosis.^1^ The involvement of surgeons with sufficient expertise is essential to ensure complete excision of lesions and to minimize the risk of reoperation and complications.^2^, ^3^, ^4^, ^5^ Preoperative transvaginal ultrasound (TVS) and magnetic resonance imaging (MRI) may facilitate optimal multidisciplinary surgical planning and improve the choice of surgical technique, but selecting the optimal diagnostic pathway in endometriosis surgery planning remains challenging.^6^ Both expert TVS and MRI demonstrate high diagnostic performance for DE, and recent prospective studies have shown largely comparable performance between the modalities.^7^, ^8^, ^9^, ^10^ However, most studies have evaluated TVS and MRI independently or as alternative diagnostic tests, while evidence on the added diagnostic contribution of MRI when used selectively as a second‐line examination after expert TVS remains limited. It remains unclear at which anatomical sites, and in which patient subgroups, second‐line MRI provides additional diagnostic information beyond expert TVS.^11^, ^12^

The primary aim of this study was to evaluate the added diagnostic yield of MRI following expert TVS in cases with suspected advanced disease, inconclusive findings, or negative TVS with typical symptoms in the preoperative assessment of DE. As an exploratory measure of clinical utility, we also assessed whether additional MRI findings were associated with changes in multidisciplinary surgical planning. The secondary aim was to assess the diagnostic performance of TVS alone using the International Deep Endometriosis Analysis (IDEA) approach.

## 2 MATERIAL AND METHODS

This prospective observational cohort study was conducted at Stockholm South General Hospital, a tertiary referral center for advanced gynecologic ultrasound in Stockholm, Sweden. The unit performs approximately 3600 ultrasound examinations annually, including 950 dedicated endometriosis assessments. A multidisciplinary surgical team (gynecologic, urologic, and colorectal surgeons) is available. Data collection was planned prospectively, before the index tests and surgical reference standard were performed. Potentially eligible patients were identified among women referred to our tertiary ultrasound unit for evaluation of suspected endometriosis between January 2019 and May 2022. They were recruited as a nonconsecutive series of eligible patients at their initial ultrasound visit. TVS and surgery were performed at the same institution. MRI was performed locally for patients included in the paired analysis; five externally performed MRI examinations were unavailable for review and were excluded from the analysis.

All patients were examined with TVS and referred for further evaluation with MRI as part of routine clinical practice. Indications for MRI were (1) suspected advanced or complex DE on TVS, (2) inconclusive examination due to suboptimal imaging conditions, or (3) symptoms suggestive of advanced endometriosis despite negative TVS findings. Accordingly, the target population for the paired TVS‐MRI analysis was patients selected for second‐line MRI within the preoperative diagnostic pathway, rather than an unselected population with suspected endometriosis or all patients undergoing surgery.

Data were collected and managed using Research Electronic Data Capture (REDCap) hosted at Karolinska Institutet. Inclusion criteria were age ≥18 years, symptoms suggestive of endometriosis, referral for TVS at our tertiary endometriosis center and planned surgery. All participants provided their written consent.

Ultrasound was performed in the lithotomy position using a Voluson E10 system (General Electric Medical Systems, Zipf, Austria) with a 5–9 MHz transvaginal transducer. No bowel preparation or contrast gel was used. Seven examiners performed acquisition, interpretation, and reporting; none were blinded to clinical data or prior imaging. All had >5 years' experience in endometriosis imaging. TVS followed the standardized IDEA approach^13^ for systematic TVS assessment summarized as: (1) assessment of the uterus and adnexa, including identification of endometriomas; (2) evaluation of soft markers, such as site‐specific tenderness and the “sliding sign” (to assess organ mobility and pouch of Douglas obliteration); (3) systematic assessment of the anterior and posterior compartments, including the bladder, uterosacral ligaments, rectovaginal septum, and bowel. Ureters were systematically assessed as part of an extended evaluation beyond the core IDEA 2016 protocol, in line with later developments in parametrial assessment. Following TVS, the examining gynecologist prospectively recorded whether colorectal or urologic surgical availability was considered necessary for the planned procedure. To assess the distribution of TVS performance across operators, case volume and site‐specific sensitivity and specificity were calculated separately for each of the seven examiners in the full surgical cohort. Examiner level analyses were descriptive because of the unequal and, for some examiners, small case volumes.

MRI was performed on a 1.5‐T scanner (Siemens, Erlangen, Germany) using a standardized protocol with multiplanar T2‐ and T1‐weighted fat‐suppressed sequences before and after gadolinium. Patients fasted for 4 h and received antiperistaltic agents before examination. Examinations were conducted in routine practice and reviewed for quality assurance by a senior radiologist with >10 years' experience (Table S1 for protocol).

The examinations were performed as part of routine clinical care, and the ultrasound examiners were not blinded to available clinical information or previous imaging findings. MRI was performed as a second‐line examination, and the radiologist had access to clinical information and the preceding TVS findings. Thus, the imaging pathway reflected a pragmatic sequential diagnostic approach rather than an independent blinded assessment of the two modalities.

Surgical visualization was chosen as the primary reference standard because it allowed direct site‐specific assessment of disease at the time of surgery. Immediately following surgery, the operating surgeon prospectively recorded the presence or absence of endometriotic involvement at predefined anatomical sites using a structured REDCap surgical form (Table S2). The anatomical sites corresponded to those assessed by preoperative imaging, including the bowel, uterosacral ligaments, urinary bladder, ureters, and pouch of Douglas. Deep endometriosis was defined as a lesion judged to infiltrate ≥5 mm beneath the tissue surface. Ovaries, bowel, uterosacral ligament, rectovaginal, bladder, and ureteral involvement were recorded separately. Pouch of Douglas obliteration was classified according to the surgeon's intraoperative visual assessment as present or absent. Adhesions alone were not considered equivalent to deep endometriosis. Surgeons were not blinded to preoperative imaging findings, as these were available for routine surgical planning.

Histopathology was assessed as a secondary reference standard when tissue from the respective anatomical site was available. Not all suspected lesions were excised or biopsied. For the site‐specific histopathological analysis, a site was classified as positive if endometriosis was confirmed in at least one specimen from that anatomical site.

Surgery was performed laparoscopically, robot assisted, or open, with findings recorded by experienced gynecologic surgeons. Procedures included salpingectomy, oophorectomy, hysterectomy, excision of lesions, bowel shaving, discoid and segmental resections, bladder lesion excision, and ureteral reimplantation. Following surgery, the involvement of colorectal and urologic surgeons during the procedure was documented. These data were used in an exploratory analysis to assess the potential relevance of additional MRI findings to multidisciplinary surgical planning. For patients in whom MRI correctly identified surgically confirmed bowel DE that had been missed by TVS, and colorectal surgical involvement had not initially been considered necessary after TVS, the available multidisciplinary team documentation was reviewed to determine whether the MRI findings had resulted in a documented change in surgical planning.

*Microsoft 365 Copilot* (Microsoft, Redmond, Washington, USA) and ChatGPT (OpenAI, San Francisco, California, USA) were used to assist with language editing and correcting STATA syntax. During the revision of this manuscript, the authors used Claude Sonnet 5 (Anthropic, San Francisco, California, USA; accessed September 2026) to improve the language of selected passages, including parts of the Discussion. All content was reviewed and approved by the authors, who take full responsibility for the final manuscript.

### 2.1 Statistical analyses

Categorical variables are presented as frequencies and percentages. Agreement between modalities was assessed using Cohen's κ and interpreted according to the Altman criteria.^14^ Diagnostic performance, including sensitivity, specificity, positive and negative predictive values (PPV and NPV), and likelihood ratios, was calculated with corresponding 95% confidence intervals (CI) for TVS and MRI across anatomical sites.

For both TVS and MRI, each anatomical site was classified as positive or negative for endometriosis according to the prospectively recorded imaging assessment. Indeterminate imaging findings were not analyzed as a separate category. For the combined imaging approach, a finding at a given anatomical site was considered positive if either TVS or MRI was positive (a predefined OR‐positive rule chosen to maximize sensitivity). By construction, this rule can only maintain or increase the number of positive findings compared with TVS alone, so sensitivity cannot decrease and specificity cannot increase. The combined approach is therefore an analytical combination rule and does not reflect how the two modalities were weighed against each other in clinical practice. The added diagnostic yield of second‐line MRI was operationally defined as the change in diagnostic performance when MRI findings were added to expert TVS under this rule. This was assessed by changes in sensitivity and specificity, together with the corresponding absolute numbers of additional true‐positive and false‐positive findings at each anatomical site. Because TVS and combined imaging were compared within the same anatomical sites, these changes were calculated from the paired findings, that is, the proportion of surgically confirmed lesions (sensitivity) or lesion‐free sites (specificity) whose classification changed from negative to positive when MRI was added. Exact binomial 95% confidence intervals were calculated for these proportions because of the small cell counts.

Missing data were handled by complete‐case analysis.

Baseline and surgical disease characteristics were compared descriptively between surgical patients who did and did not undergo second‐line MRI to characterize selection into the MRI subgroup. No formal statistical comparisons were performed, as these analyses were intended to describe the selected study population rather than to test differences between groups.

Subgroup analyses according to previous surgical status and BMI‐category (<25, 25–29.9, and ≥30) were performed using a two‐sided Fisher's exact test. Given the limited subgroup sample sizes, these analyses were considered hypothesis‐generating and exploratory. No formal sample size calculation was performed.

Statistical analyses were conducted using Stata version 18 (StataCorp, College Station, Texas, USA).

## 3 RESULTS

221 of 222 asked patients consented to participate. Of these, 189 underwent surgery, whereas 32 received nonsurgical management (Figure 1). Of the 112 cases referred to MRI, 107 were eligible for analysis, having undergone TVS, had available MRI scans, and went through surgery. Baseline and surgical characteristics of patients undergoing surgery with and without second‐line MRI are presented in Table 1. Patients selected for second‐line MRI had a substantially higher prevalence of surgically confirmed endometriosis across all anatomical sites assessed, particularly bowel DE and obliteration of the pouch of Douglas. These differences reflect the selective use of MRI according to clinical indication and characterize the population to which the paired TVS‐MRI analysis applies. Among patients undergoing surgery, 135/189 (71%) had robot‐assisted laparoscopy, 52/189 (28%) conventional laparoscopy, and 2/189 (1%) laparotomy, with no conversions between approaches. The mean time from TVS to surgery was 115 days. Surgically visualized endometriosis was present in 165/189 (87%) patients, of whom 155/165 (94%) had histologic confirmation.

**FIGURE 1:**
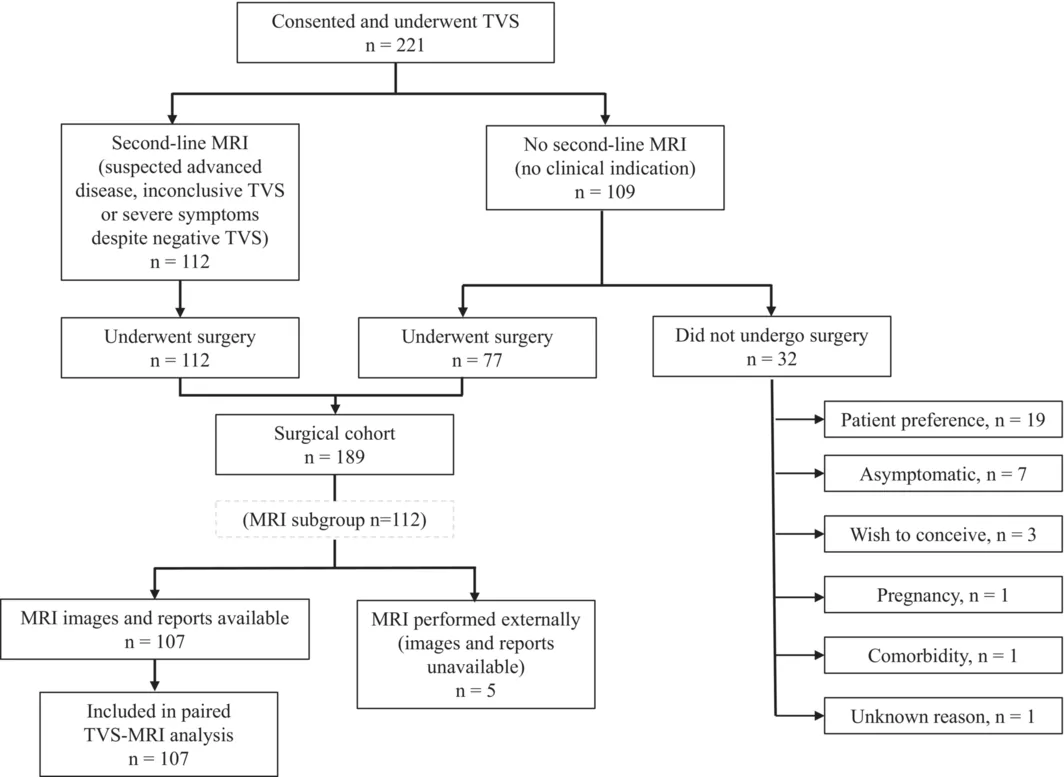
Flowchart of study participants. *n* = number, TVS = transvaginal ultrasound, MRI = magnetic resonance imaging.

**TABLE 1:** Clinical and surgical characteristics according to second‐line MRI.

Characteristic	No second‐line MRI (*n* = 77)	Second‐line MRI (*n* = 112)
Age, years	33.2 ± 10.6	40.3 ± 8.1
BMI, kg/m^2^	23.9 ± 3.8	27.1 ± 6.1
Parity		
0	51/77 (66.2%)	57/112 (50.9%)
1–2	25/77 (32.5%)	47/112 (42.0%)
>2	1/77 (1.3%)	8/112 (7.1%)
Infertility	47/74 (63.5%)	82/104 (78.8%)
Previous abdominal surgery	27/73 (37.0%)	57/111 (51.4%)
Surgery due to endometriosis	14/27 (51.9%)	37/57 (64.9%)
Cesarean section	9/27 (33.3%)	16/57 (28.1%)
Other surgery	10/27 (37.0%)	22/56 (39.3%)
Spontaneous menstrual cycle	32/77 (41.6%)	52/111 (46.8%)
Hormonal treatment		
Combined oral contraceptive	18/77 (23.4%)	14/112 (12.5%)
Progestin	13/77 (16.9%)	27/112 (24.1%)
LNG‐IUS	19/77 (24.7%)	14/112 (12.5%)
GnRH analog	1/77 (1.3%)	8/112 (7.1%)
Aromatase inhibitor	1/77 (1.3%)	0/112 (0.0%)
Symptoms		
Abnormal bleeding	18/77 (23.4%)	13/112 (11.6%)
Hematuria	1/77 (1.3%)	1/109 (0.9%)
Hematochezia	7/76 (9.2%)	7/111 (6.3%)
Dysmenorrhea	70/77 (90.9%)	100/112 (89.3%)
Dysuria	32/76 (42.1%)	54/112 (48.2%)
Dyschezia	40/75 (53.3%)	64/112 (57.1%)
Dyspareunia	46/75 (61.3%)	62/108 (57.4%)
Chronic pelvic pain	30/76 (39.5%)	55/111 (49.5%)
Surgical disease characteristics		
Endometrioma	27/77 (35.1%)	88/112 (78.6%)
Bowel DE	3/77 (3.9%)	61/112 (54.5%)
Uterosacral ligament DE	9/77 (11.7%)	49/112 (43.8%)
Urinary tract DE	0/77 (0.0%)	21/112 (18.8%)
Obliterated pouch of Douglas	6/77 (7.8%)	64/112 (57.1%)

*Note*: Data are presented as mean ± SD or *n*/*N* (%), unless otherwise indicated. Missing values were excluded from the denominator. The second‐line MRI group includes five patients who underwent MRI at an external hospital but whose MRI images and reports were unavailable for the paired diagnostic analysis.

Abbreviations: BMI, body mass index; DE, deep endometriosis; GnRH, gonadotropin‐releasing hormone; LNG‐IUS, levonorgestrel‐releasing intrauterine system; SD, standard deviation.

Table 2 shows the diagnostic performance of TVS and the combined approach (TVS + MRI) using surgical visualization as the reference standard (full data in Table S3, histolog reference in Table S4). A combined positive result was defined as a positive finding on either modality. The absolute changes in sensitivity and specificity after the addition of MRI, together with the corresponding changes in true positive and true negative findings, are illustrated in Figure 2.

**TABLE 2:** Diagnostic performance of transvaginal ultrasound and combined transvaginal ultrasound with magnetic resonance imaging in detecting deep endometriosis and endometriomas with surgical visualization as reference standard. A combined test result was defined as positive when at least one modality was positive and negative only when both modalities were negative, *n* = 107.

		Sens% 95% CI	Δ Sens% 95% CI	Spec% 95% CI	Δ Spec % 95% CI	PPV% 95% CI	NPV% 95% CI	TP n	FP n	TN n	FN n
Endometrioma *n* = 86 Prev 80.4% 95% CI 71.6–87.4	TVS	96.5 90.1–99.3	+2.3 0.3–8.1	85.7 63.7–97.0	−19.0 −41.9 to −5.4	96.5 90.1–99.3	85.7 63.7–97.0	83	3	18	3
	TVS + MRI	98.8 93.7–100.0		66.7 43.0–85.4		92.4 84.9–96.9	93.3 68.1–99.8	85	7	14	1
Urinary tract (urinary bladder and ureters) *n* = 21 Prev 19.6% 95% CI 12.6–28.4	TVS	42.9 21.8–66.0	+4,8 0.1–23.8	91.9 83.9–96.7	−9.3 −17.5 to −4.1	56.3 29.9–80.2	86.8 78.1–93.0	Bladder: 1 Ureters: 8 Any: 9	2 5 7	101 82 79	3 12 12
	TVS + MRI	47.6 25.7–70.2		82.6 72.9–89.9		40.0 21.1–61.3	86.6 77.3–93.1	Bladder: 2 Ureters: 9 Any: 10	7 8 15	96 79 71	2 11 11
Bowel *n* = 58 Prev 54.2% 95% CI 44.3–63.9	TVS	81.0 68.6–90.1	+10.3 3.9–21.2	61.2 46.2–74.8	−6.1 −16.9 to −1.3	71.2 58.7–81.7	73.2 57.1–85.8	47	19	30	11
	TVS + MRI	91.4 81.0–97.1		55.1 40.2–69.3		70.7 59.0–80.6	84.4 67.2–94.7	53	22	27	5
USLs *n* = 48 Prev 44.9% 95% CI 35.2–54.8	TVS	58.3 43.2–72.4	+29.2 17.0–44.1	59.3 45.7–71.9	−20.3 −32.8 to −11.0	53.8 39.5–67.8	63.6 49.6–76.2	28	24	35	20
	TVS + MRI	87.5 74.8–95.3		39.0 26.5–52.6		53.8 42.2–65.2	79.3 60.3–92.0	42	36	23	6
Obliterated POD *n* = 62 Prev 57.9% 95% CI 48.0–67.4	TVS	98.4 91.3–100.0	+1.6 0.0–8.7	31.1 18.2–46.6	−13.3 −26.8 to −5.1	66.3 55.7–75.8	93.3 68.1–99.8	61	31	14	1
	TVS + MRI	100 94.2–100.0		17.8 8.0–32.1		62.6 52.3–72.1	100 63.1–100.0	62	37	8	0

Abbreviations: Δ, delta, differences; CI, confidence interval; FN, false negative; MRI, magnetic resonance imaging; POD, pouch of Douglas; PPV, positive predictive value; Prev, prevalence; Sens, sensitivity; Spec, specificity; TN, true negative; TP, true positive; TVS, transvaginal ultrasound; USL, uterosacral ligaments.

**FIGURE 2:**
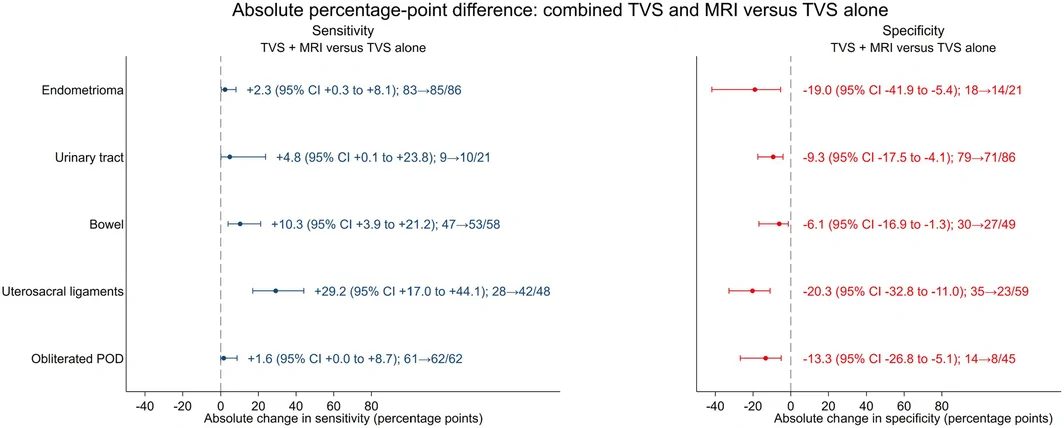
Change in diagnostic performance with the addition of magnetic resonance imaging to transvaginal ultrasound. Differences are expressed as percentage‐point changes for the combined TVS + MRI approach compared with TVS alone, with exact binomial 95% confidence intervals. Numbers shown for sensitivity represent true‐positive findings among patients with surgically confirmed disease (TP/*n*), and numbers shown for specificity represent true‐negative findings among patients without surgically confirmed disease (TN/*n*). The combined approach was considered positive when either TVS or MRI was positive. Dots represent absolute differences; horizontal lines represent 95% confidence intervals. POD, pouch of Douglas.

Among patients assessed with both modalities, 11/58 bowel DE cases were missed by TVS, 10 located in the mid or upper rectum. Of these, MRI detected 6/11 (55%), all in the upper rectum or rectosigmoid. All missed lesions occurred in patients with additional DE in other locations and were classified as advanced disease. Among the 11 bowel lesions missed by TVS, colorectal surgical assistance was present in eight cases. All 11 bowel lesions were managed with bowel shaving; none underwent discoid, full‐thickness, or segmental bowel resection. TVS identified bowel lesions not seen on MRI in eight cases, often due to misclassification as adhesions on MRI; the same mechanism explained five cases missed by both modalities.

In an exploratory assessment of the potential clinical relevance of additional MRI findings, nine patients received colorectal surgical assistance despite this not having been considered necessary after TVS. Seven had surgically confirmed bowel DE. In three of these seven patients, bowel involvement was missed by TVS but correctly identified by MRI. Targeted review of the multidisciplinary team documentation for these three cases showed that, following MRI, the surgical plan was revised to include colorectal surgical participation in all three. In comparison, among 12 patients who received urological assistance despite this not having been considered necessary after TVS, five had surgically confirmed urinary tract DE. MRI did not identify any additional true‐positive urinary tract lesions missed by TVS; four of the five surgically confirmed lesions were missed by both modalities.

Diagnostic performance varied across anatomical locations and according to prior surgical status (see Table 3). For endometriomas, both TVS and the combined approach demonstrated high sensitivity (>94%) regardless of surgical history, with no significant differences in sensitivity or specificity between groups. In exploratory subgroup analyses, sensitivity of the combined TVS‐MRI approach for bowel DE was higher among women with previous abdominal surgery than among those without previous surgery (100%, 35/35, vs. 77.3%, 17/22; Fisher's exact *p* = 0.006). No significant differences in specificity were observed between groups. No corresponding difference was observed for TVS alone (85.7%, 30/35, vs. 72.7%, 16/22; *p* = 0.305). Given the small subgroup sizes, these findings should be considered hypothesis‐generating. For urinary tract DE, TVS specificity was numerically higher among women without previous abdominal surgery than among those with previous surgery (97.7%, 42/43, vs. 85.7%, 36/42), although the difference was not statistically significant using Fisher's exact test (*p* = 0.058). Stratification by BMI category revealed no significant differences in sensitivity or specificity for either TVS or the combined method.

**TABLE 3:** Diagnostic performance of transvaginal ultrasound and combined method (transvaginal ultrasound and magnetic resonance imaging) in detecting deep endometriosis and endometriomas according to surgical history with surgical findings as reference standard, *n* = 106.

		Sensitivity%			Specificity%		
		Prior abdominal surgery *n* = 55 (95% CI)	No prior abdominal surgery *n* = 51 (95% CI)	Fisher's exact test on surgery status *p*‐value	Prior abdominal surgery *n* = 55 (95% CI)	No prior abdominal surgery *n* = 51 (95% CI)	Fisher's exact test on surgery status *p*‐value
Endometrioma *n* = 85 (106)	TVS	94.9 (82.7–99.4)	97.8 (88.5–99.9)	0.591	93.8 (69.8–99.8)	60.0 (14.7–94.7)	0.128
	TVS + MRI	97.4 (86.5–99.9)	100.0 (92.3–100.0)	0.459	75.0 (47.6–92.7)	40.0 (5.3–85.3)	0.280
Urinary tract (urinary bladder and ureters) *n* = 21 (106)	TVS	46.2 (19.2–74.9)	37.5 (8.5–75.5)	1.0	85.7 (71.5–94.6)	97.7 (87.7–99.9)	0.058
	TVS + MRI	46.2 (19.2–74.9)	50.0 (15.7–84.3)	1.0	76.2 (60.5–87.9)	88.4 (74.9–96.1)	0.165
Bowel *n* = 57 (106)	TVS	85.7 (69.7–95.2)	72.7 (49.8–89.3)	0.305	60.0 (36.1–80.9)	62.1 (42.3–79.3)	1.0
	TVS + MRI	100.0 (90.0–100.0)	77.3 (54.6–92.2)	0.006	55.0 (31.5–76.9)	55.2 (35.7–73.6)	1.0
USLs *n* = 48 (106)	TVS	50.0 (29.1–70.9)	66.7 (44.7–84.4)	0.380	58.1 (39.1–75.5)	59.3 (38.8–77.6)	1.0
	TVS + MRI	83.3 (62.6–95.3)	91.7 (73.0–99.0)	0.666	38.7 (21.8–57.8)	37.0 (19.4–57.6)	1.0

Abbreviations: CI, confidence interval; MRI, magnetic resonance imaging; *n*, number; TVS, transvaginal ultrasound; USLs, uterosacral ligaments.

Table 4 shows the diagnostic performance of TVS and MRI as individual modalities using surgical visualization as the reference standard (*n* = 107). Overall, accuracy was higher for TVS than MRI across most locations, except for bowel lesions where MRI showed slightly higher accuracy.

**TABLE 4:** Diagnostic performance of transvaginal ultrasound and magnetic resonance imaging in detecting deep endometriosis and endometriomas with surgical findings as reference standard, *n* = 107.

		Accuracy%	Prev%, 95% CI	Cohen's kappa, *κ* (std. error)	Sens% 95% CI	Spec% 95% CI	PPV% c	NPV%95% CI	LR+ *95% CI*	LR− 95% CI	TP *n*	FP *n*	TN *n*	FN *n*
Adnexa														
Endometrioma	TVS	94.4 88.0–97.5	80.4 71.6–87.4	0.82 (0.097)	96.5 90.1–99.3	85.7 63.7–97.0	96.5 90.1–99.3	85.7 63.7–97.0	6.76 2.37–19.28	0.04 0.01–0.13	83	3	18	3
	MRI	88.8 81.2–93.6	80.4 71.6–87.4	0.65 (0.097)	93.0 85.4–97.4	71.4 47.8–88.7	93.0 85.4–97.4	71.4 47.8–88.7	3.26 1.65–6.42	0.10 0.04–0.22	80	6	15	6
Anterior compartment and ureters														
Urinary tract (urinary bladder and ureters)	TVS	82.2 73.7–88.4	19.6 12.6–28.4	0.38 (0.095)	42.9 21.8–66.0	91.9 83.9–96.7	56.2 29.9–80.2	86.8 78.1–93.0	5.27 2.22–12.50	0.62 0.43–0.91	Bladder: 1 Ureters: 8 Any: 9	2 5 7	101 82 79	3 12 12
	MRI	78.5 69.6–85.3	19.6 12.6–28.4	0.28 (0.096)	38.1 18.1–61.6	88.4 79.7–94.3	44.4 21.5–69.2	85.4 76.3–92.0	3.28 1.48–7.28	0.70 0.50–0.99	Bladder: 2 Ureters: 7 Any: 8	5 5 10	98 82 76	2 13 13
Posterior compartment														
Bowel	TVS	72.0 62,6–79.7	54.2 44.3–63.9	0.42 (0.095)	81.0 68.6–90.1	61.2 46.2–74.8	71.2 58.7–81.7	73.2 57.1–85.8	2.10 1.44–3.04	0.31 0.17–0.55	47	19	30	11
	MRI	73.8 64.6–81.4	54.2 44.3–63.9	0.47 (0.096)	77.6 64.7–87.5	69.4 54.6–81.7	75.0 62.1–85.3	72.3 57.4–84.4	2.53 1.63–3.95	0.32 0.19–0.54	45	15	34	13
USLs	TVS	58.9 49.2–67.9	44.9 35.2–54.8	0.17 (0.096)	58.3 43.2–72.4	59.3 45.7–71.9	53.8 39.5–67.8	63.6 49.6–76.2	1.43 0.97–2.12	0.70 0.47–1.04	28	24	35	20
	MRI	52.3 42.8–61.7	44.9 35.2–54.8	0.04 (0.096)	50.0 35.2–64.8	54.2 40.8–67.3	47.1 32.9–61.5	57.1 43.2–70.3	1.09 0.73–1.62	0.92 0.64–1.33	24	27	32	24
Obliterated POD	TVS	70.1 60.7–78.1	57.9 48.0–67.4	0.32 (0.074)	98.4 91.3–100.0	31.1 18.2–46.6	66.3 55.7–75.8	93.3 68.1–99.8	1.43 1.17–1.74	0.05 0.01–0.38	61	31	14	1
	MRI	58.9 49.2–67.9	57.9 48.0–67.4	0.15 (0.096)	66.1 53.0–77.7	48.9 33.7–64.2	64.1 51.1–75.7	51.2 35.5–66.7	1.29 0.92–1.81	0.69 0.44–1.10	41	23	22	21

Abbreviations: CI, confidence interval; FN, false negative; LR+, positive likelihood ratio; LR−, negative likelihood ratio; MRI, magnetic resonance imaging; *n*, number; NPV, negative predictive value; POD, pouch of Douglas; PPV, positive predictive value; Prev, prevalence; Sens, sensitivity; Spec, specificity; TN, true negative; TP, true positive; TVS, transvaginal ultrasound; USL, uterosacral ligaments.

Case volume and site‐specific diagnostic performance of TVS for each of the seven sonologists are presented in Table S5. Case volumes were unevenly distributed, ranging from 3 to 121 examinations per sonologist. Examiner‐specific estimates varied across anatomical sites; however, interpretation of these differences was limited by the small number of examinations and disease positive or disease negative cases for several examiners. Among the 77 patients who did not undergo MRI, bowel endometriosis was missed by TVS in three cases (4%), of which two were histologically confirmed; both histologically confirmed lesions were located in the rectum (6–10 cm and >10 cm from the anal verge). Uterosacral ligament lesions were missed by TVS in eight cases (10%; 8/77), while no cases of ureteral or bladder involvement were missed.

The diagnostic performance of TVS for the full cohort is presented in Table 5 (*n* = 189), using surgical findings as the reference standard. Positive and negative predictive values were high for endometriomas (90.9% and 92.6%), and the negative predictive value was high for pouch of Douglas obliteration (94.3%).

**TABLE 5:** Performance of transvaginal ultrasound in detecting deep endometriosis in different anatomical sites of the abdomen with surgical findings as reference standard for the whole cohort, *n* = 189.

	Accuracy%, 95% CI	Prev% 95%CI	Sens% 95% CI	Spec% 95% CI	PPV% 95% CI	NPV% 95% CI	LR+ 95% CI	LR− 95% CI	TP *n*	FP *n*	TN *n*	FN *n*
Adnexa												
Endometrioma *n* = 115	91.5 86.6–94.8	60.8 53.5–67.8	95.7 90.1–98.6	85.1 75.0–92.3	90.9 84.3–95.4	92.6 83.7–97.6	6.43 3.72–11.12	0.05 0.02–0.12	110	11	63	5
Anterior compartment												
Urinary tract (urinary bladder and ureters) *n* = 21 (187)	89.8 84.6–93.4	11.2 7.1–16.7	42.9 21.8–66.0	95.8 91.5–98.3	56.2 29.9–80.2	93.0 88.1–96.3	10.16 4.23–24.43	0.60 0.41–0.87	Bladder: 1 Ureters: 8 Any: 9	2 5 7	183 162 159	3 12 12
Posterior compartment												
Bowel *n* = 64	82.0 75.8–86.9	33.9 27.2–41.1	78.1 66.0–87.5	84.0 76.4–89.9	71.4 59.4–81.6	88.2 81.0–93.4	4.88 3.20–7.45	0.26 0.16–0.42	50	20	105	14
USL *n* = 58	71.4 64.5–77.5	30.7 24.2–37.8	51.7 38.2–65.0	80.2 72.3–86.6	53.6 39.7–67.0	78.9 71.0–85.5	2.61 1.70–3.98	0.60 0.46–0.80	30	26	105	28
Obliterated POD *n* = 70	77.8 71.3–83.2	37.0 30.1–44.3	92.9 84.1–97.6	68.9 59.8–77.1	63.7 53.6–73.0	94.3 87.1–98.1	2.99 2.27–3.93	0.10 0.04–0.24	65	37	82	5

Abbreviations: CI, confidence interval; FN, false negative; LR−, negative likelihood ratio; LR+, positive likelihood ratio; n, number; NPV, negative predictive value; POD, pouch of Douglas; PPV, positive predictive value; Prev, prevalence; Sens, sensitivity; Spec, specificity; TN, true negative; TP, true positive; USL, uterosacral ligament.

## 4 DISCUSSION

In this prospective cohort study, we assessed the added diagnostic yield of MRI as a second‐line test after expert TVS in women who underwent MRI for suspected advanced DE, inconclusive TVS findings, or persistent clinical suspicion despite negative TVS. The most clinically relevant finding was the improved sensitivity for bowel involvement when MRI was added to TVS, particularly in patients with previous abdominal surgery. Most lesions missed by TVS were located in the mid or upper rectum, highlighting a known anatomical limitation of TVS. Some false positive bowel findings resulted from misclassification of uterosacral ligament lesions as bowel involvement. Recent data suggest that complex posterior compartment disease can influence the diagnostic assessment of uterosacral ligament and torus uterinus endometriosis, underscoring the importance of careful anatomical interpretation in advanced disease.^15^ Our findings suggest that MRI may be particularly helpful in complex cases where altered anatomy or adhesions could limit ultrasound assessment.

For urinary tract involvement, sensitivity remained low for both modalities. TVS specificity was numerically higher in patients without prior surgery, while the addition of MRI increased false positive findings irrespective of previous surgical status. Among patients who received urologic assistance although it had not been considered necessary after TVS, MRI identified no additional true positive urinary tract lesions. No meaningful improvement was observed for endometriomas, where TVS already demonstrated high diagnostic performance, consistent with previous literature.^16^

Accurate assessment of lesion extent is important for preoperative planning and surgical team allocation. For bowel and uterosacral ligament disease, in particular, the higher sensitivity achieved with the combined approach came at the cost of lower specificity and an increased rate of false positive findings, which in a preoperative setting risks overestimating disease extent and may lead to unnecessary multidisciplinary planning, altered patient counseling, or a perceived need for more complex surgery. Taken together, our findings suggest that MRI may have a role as a selective second‐line investigation for refining disease mapping in complex cases where clinically relevant disease remains suspected after expert TVS. Whether this additional diagnostic information justifies the organizational effort and healthcare resources required for MRI cannot be determined from the present study. In settings with flexible surgical resources, the added value of MRI may be limited, whereas in centers requiring predefined multidisciplinary planning, MRI may aid decision‐making despite false positives.

Most diagnostic accuracy studies in DE have evaluated TVS and MRI as independent modalities. Although some studies have assessed combined approaches, prospective data on sequential strategy in suspected advanced or inconclusive cases, using the IDEA consensus methodology remain limited.^9^, ^11^, ^17^, ^18^, ^19^, ^20^ Roditis et al. conducted a retrospective accuracy study concluding that combining physical examination, TVS, and MRI in diagnosing DE is more accurate than each technique separately.^18^ Alborzi et al. compared the value of MRI and TVS in the assessment of rectal endometriosis in 555 patients and found that the modalities performed almost identically in deep rectal lesions.^19^ More recently, a large prospective head‐to‐head study using the IDEA protocol and #Enzian classification found comparable diagnostic performance of ultrasound and MRI across most anatomical compartments.^7^

Our findings are broadly consistent with previous studies reporting high diagnostic accuracy of TVS for DE. Overall sensitivity and specificity in our cohort (97% and 86%) were comparable to those reported by Nisenblat et al. and Leonardi et al.^6^, ^21^ as well as the large prospective IDEA study by Bafort et al., which demonstrated similar performance (95% sensitivity and 80% specificity).^22^ Consistent with previous studies, TVS showed high accuracy for endometriomas and pouch of Douglas obliteration, while performance for uterosacral ligament lesions remained moderate, reflecting the known difficulty of assessing this location.^21^

The strengths of this study include its prospective design and thorough data collection. The examiners and surgeons recorded and staged the patients themselves, with documentation performed immediately after examination or surgery, reducing the risk of recall bias. The involvement of multiple examiners and surgeons reflects real‐world clinical practice and enhances the generalizability of the findings. The coverage of histologic confirmation was high.

This study has several limitations. The imaging assessments were not performed independently or under blinded conditions. MRI was interpreted with knowledge of the preceding TVS findings, reflecting the sequential diagnostic pathway used in clinical practice. This may have introduced review bias and should be considered when interpreting the diagnostic performance of MRI and the combined imaging approach. Previous abdominal surgery may have introduced additional bias, both because examiners were aware of the patients' surgical history and because postoperative adhesions and fibrosis may have altered the pelvic anatomy and affected imaging assessment. However, previously operated patients were retained because they represent an important part of the population undergoing assessment for suspected recurrent or persistent DE in clinical practice. Their inclusion also allowed diagnostic performance to be explored separately in women with and without previous abdominal surgery.

This paired TVS‐MRI cohort is a high‐risk population enriched for more extensive disease, as reflected by the substantially higher prevalence of surgically confirmed disease across anatomical sites than in the surgical cohort as a whole. This selection may introduce spectrum bias and limits the generalizability of the diagnostic performance estimates to unselected populations with suspected endometriosis. Since we evaluated diagnostic performance in a high‐prevalence tertiary referral population, similar to other published studies with a high prevalence of DE,^9^, ^17^, ^18^ this may have led to overestimation of predictive values. This reflects referral patterns in which MRI is reserved for patients with high suspicion of disease. The high prevalence likely introduced a ceiling effect, limiting additional gains in diagnostic accuracy and partly explaining the limited incremental benefit for some sites.

The comparison between TVS and combined TVS + MRI was inherently nonindependent since the combined approach incorporated the TVS findings. Because MRI was selectively performed, its diagnostic performance could not be assessed in the full surgical cohort, and potential MRI findings among patients not referred for MRI remain unknown. Furthermore, case volume was unevenly distributed across the seven examiners, with the majority of examinations performed by one examiner. Although examiner‐specific diagnostic performance is provided, the small case volumes for several examiners precluded meaningful comparisons between operators. The sample size was moderate, limiting subgroup analyses. Vaginal fornix and rectovaginal septum lesions could not be evaluated due to low numbers (*n* = 2). No formal sample size calculation was performed, and several site‐specific and subgroup‐specific estimates were associated with limited precision. Confidence intervals should therefore be considered when interpreting the magnitude of observed differences.

The study period preceded recent advances in ultrasound assessment of DE in the lateral pelvic compartment, including parametrial and pelvic sidewall evaluation, as described in IDEA consensus addenda 2024 and 2025.^23^, ^24^ Emerging techniques for visualization of the sacral plexus have also been introduced more recently.^25^ These locations were not systematically assessed in the present study. Thus, this study does not evaluate contemporary comprehensive DE mapping, but rather the sequential imaging pathway and anatomical assessment used during the study period (2019–2022). The findings may therefore not be fully generalizable to centers currently applying updated IDEA recommendations or #Enzian‐based systematic mapping. However, ureteral involvement was systematically evaluated, including assessment of ureteral narrowing, obstruction, and hydronephrosis, in accordance with clinical practice at the time. Last, this study was not designed to determine whether the addition of MRI translates into improved multidisciplinary composition or postoperative outcomes. Although MRI findings influenced multidisciplinary surgical planning in selected cases, we cannot determine whether this resulted in improved surgical or patient‐reported outcomes.

## 5 CONCLUSION

In selected complex patients undergoing second‐line MRI after expert TVS, combined imaging increased detection of bowel and uterosacral ligament lesions, but reduced specificity at several anatomical sites. The sensitivity appeared greater among previously operated patients, although this subgroup finding is exploratory. These results are consistent with a selective rather than routine role for MRI.

## AUTHOR CONTRIBUTIONS

Malin Brunes and Elisabeth Epstein conceived and designed the study. Mira Rehal, Malin Brunes, and Elisabeth Epstein recruited patients, collected the data, analyzed, and interpreted the data. Mira Rehal performed the statistical analysis in consultation with a statistician. Malin Brunes and Elisabeth Epstein were among the examiners who performed the ultrasound examinations, Chikako Suzuki reviewed the MRI images, and Malin Brunes was part of the surgical team. Mira Rehal was primarily responsible for writing the manuscript, including the first draft, and Elisabeth Epstein, Malin Brunes, and Chikako Suzuki critically revised it. All authors approved the final version.

## FUNDING INFORMATION

The authors gratefully acknowledge the financial support provided by Insamlingsstiftelsen Kvinnor & Hälsa (founded by Alexandra – för Kvinnor & Hälsa, formerly the 1.6 and 2.6 Million Clubs) for their support. The study was supported by the Swedish Research Council (2020‐01702 and 2025‐02743), the Swedish Cancer Society (211657 Pi 01 H, 25 4609 S Tu, and 24 3381 Pj), the Stockholm Regional Council (FoUl‐954673, FoUI‐953813, FoUI‐972888, FoUI‐1028887 and FoUI‐1019922), and the Radiumhemmet Research Fund (231143, E.E.).

## CONFLICT OF INTEREST STATEMENT

The authors Mira Rehal, Malin Brunes, and Chikako Suzuki declare no conflict of interest. Elisabeth Epstein declares royalties from Studentlitteratur for a practical guidebook on ultrasound diagnostics in obstetrics and gynecology; support for attending meetings as an invited speaker at international conferences (ISUOG, ESGO, NFOG, and SFOG); a pending patent on AI‐driven diagnostic support for ultrasound assessment of ovarian tumors; a leadership role on the board of Intelligyn; and stock in Intelligyn. No payment has been made to Elisabeth Epstein or her institution for the board role.

## ETHICS STATEMENT

The study was approved by the Swedish Ethical Review Authority on August 22, 2018 (Dnr 2018/1380‐31/1), and all patients provided written informed consent.

## Supporting information


**Table S1.** Magnetic resonance imaging protocol used by radiologist in review. DE, deep endometriosis; USL, uterosacral ligament; POD, pouch of Douglas.
**Table S2.** Surgical protocol used by the operating surgeon for prospective recording of intraoperative findings. DE, deep endometriosis; USL, uterosacral ligament.
**Table S3.** Diagnostic performance of transvaginal ultrasound and combined transvaginal ultrasound with magnetic resonance imaging in detecting deep endometriosis and endometriomas with surgical visualization as the reference standard. A combined test result was defined as positive when at least one modality was positive and negative only when both modalities were negative, *n* = 107. n, number; CI, confidence interval; TVS, transvaginal ultrasonography; MRI, magnetic resonance imaging; USL, uterosacral ligament; POD, pouch of Douglas; Prev, prevalence; Sens, sensitivity; Spec, specificity; PPV, positive predictive value; NPV, negative predictive value; TP, true positive; TN, true negative; FN, false negative; LR+, positive likelihood ratio; LR−, negative likelihood ratio; Δ, delta, differences.
**Table S4.** Diagnostic performance of transvaginal ultrasound for detecting deep endometriosis and endometriomas, using histological diagnosis as the reference standard, *n* = 189. TVS = transvaginal ultrasonography, MRI = magnetic resonance imaging, USL = uterosacral ligament, POD = pouch of Douglas, Prev = prevalence, Sens = sensitivity, Spec = specificity, PPV = positive predictive value, NPV = negative predictive value, LR+ = positive likelihood ratio, LR− = negative likelihood ratio.
**Table S5.** Case volume and site‐specific diagnostic performance of TVS by individual examiner in the surgical cohort (*n* = 189). Data are presented as n/N, USL = uterosacral ligaments, POD = pouch of Douglas, NE = not estimable. Examiner‐specific estimates should be interpreted cautiously because case volumes were highly uneven across sonologists, particularly for less prevalent anatomical sites.

## Data Availability

The data that support the findings of this study are available on request from the corresponding author. The data are not publicly available due to privacy or ethical restrictions.
