# Supplementary material for: Second‐line MRI after expert transvaginal ultrasound in selected patients with suspected complex deep endometriosis: A prospective cohort study
Source: Acta Obstet Gynecol Scand. 2026 Oct 23:10.1111/aogs.70372. Online ahead of print. doi: 10.1111/aogs.70372 (PMC13691578; doi:10.1111/aogs.70372)
Supplement: Supplementary file 1 [file AOGS-9999-0-s001.docx]

Table S1. Magnetic resonance imaging protocol used by radiologist in review.

| Question | Answer alternatives |  |  |
| --- | --- | --- | --- |
| **Does the patient have a uterus?** | Yes/No. If yes   - Are there signs of adenomyosis? |  |  |
| **Right Ovary: endometrioma?** | Yes/No |  |  |
| **Right Ovary: Signs of adhesions?** | Yes/No. If yes   - Medial, lateral or both? |  |  |
| **Right Ovary: Other lesions than endometrioma** | Yes/No. If yes   - Specify the lesion |  |  |
| **Right side: Hematosalpinx?** | Yes//No | |  |
| **Right side: Hydrosalpinx?** | Yes/No | |  |
| **Left Ovary: endometrioma?** | Yes/No. If yes   - Dimensions of endometrioma? (mm) | |  |
| **Left Ovary: Signs of adhesions?** | Yes/No. If yes   - Medial, lateral or both? |  |  |
| **Left Ovary: Other lesions than endometrioma?** | Yes/No. If yes   - Specify the lesion | |  |
| **Left side: Hematosalpinx?** | Yes/No | | |
| **Left side: Hydrosalpinx?** | Yes/No | | |
| **“Kissing ovaries”?** | Yes/No | | |
| **Is the vesicouterine pouch obliterated?** | Yes/No | | |
| **How many DE lesions is seen in the anterior compartment?** | (n) | | |
| **Is there DE in the urinary bladder wall?** | Yes/No. If yes   - Localization and size of the largest lesion? - Depth of infiltration? (mm) | |  |
| **Hydroureter on the right side?** | Yes/No |  |  |
| **Hydronephrosis on the right side?** | Yes/No. If yes   - Grade? | | |
| **Hydroureter on the left side?** | Yes/No | | |
| **Hydronephrosis on the left side?** | Yes/No. If yes   - Grade? | | |
| **Is the POD obliterated?** | Yes/No. If yes   - Partly or completely? | |  |
| **DE in the rectovaginal septum** | Yes/No. If yes   - Only in septum, in the vaginal wall, rectum, hard to tell. - Dimensions of largest lesion? (mm) | |  |
| **DE on USL?** | Yes/No. If yes   - On what side? - Dimensions of largest lesion? (mm) | |  |
| **DE on torus uterinus** | Yes/No. If yes   - Dimensions of largest lesion? (mm) |  |  |
| **DE in vaginal fornix** | Yes/No. If yes   - Dimensions of largest lesion? (mm) |  |  |
| **Is the bowel affected by endometriosis?** | Yes/No. If yes   - Number of DE-lesions in the bowel? - Localisation of the largest lesion? - Dimension of the largest lesion? (mm) - Infiltration of the muscularis? (mm) - Multifocal? - Regular or irregular? - Partly or completely infiltrating the muscularis wall? | |  |
| **Is there DE in the abdominal wall?** | Yes/No. | |  |
| **DE on teres uteri ligament?** | Yes/No. | | |

DE = deep endometriosis, USL = uterosacral ligament, POD = pouch of Douglas

Table S2. Surgical protocol used by the operating surgeon for prospective recording of intraoperative findings.

| Question | Answer alternatives |
| --- | --- |
| **Was endometriosis visible intraoperatively?** | Yes/No |
| **Endometriosis location** | Endometrioma / Hematosalpinx or hydrosalpinx / Superficial endometriosis / Deep endometriosis / Endometriosis outside the pelvis |
| **Endometrioma** | Right/Left |
| → Number of endometriomas | (n) |
| → Surgical procedure | None / Drainage and ablation / Cystectomy / Salpingo-oophorectomy / Oophorectomy |
| **Superficial endometriosis anterior to the uterus** | Urinary bladder / Vesicouterine pouch / Right pelvic sidewall / Left pelvic sidewall |
| **Superficial endometriosis on the uterus** | Anterior wall / Posterior wall |
| **Superficial endometriosis on the ovaries** | Right/Left |
| **Superficial endometriosis on the fallopian tubes** | Right/Left |
| **Superficial endometriosis posterior to the uterus** | Pouch of Douglas / Right ovarian fossa / Left ovarian fossa / Right USL / Left USL |
| **Was all superficial endometriosis resected?** | Yes/No |
| **If no, why was complete resection not performed?** | Free text |
| **DE anterior to the uterus** | Urinary bladder/ureter / Adhesions / Both |
| **DE in the urinary bladder?** | Yes/No. If yes: |
| → Number of lesions | 1–3 |
| → Depth of invasion | Partial thickness / Full thickness / Not defined |
| → Were lesion dimensions recorded? | Yes/No |
| → Dimensions of lesion | X × Y × Z (mm) |
| → Surgical procedure | Serosal shaving / Partial-thickness resection / Full-thickness resection / None |
| **Ureteral DE?** | Yes/No. If yes: |
| → On what side? | Right/Left |
| → Were lesion dimensions recorded? | Yes/No |
| → Dimensions of lesion | X × Y × Z (mm) |
| → Surgical procedure | Ureterolysis / Resection / None |
| → If resected | Re-anastomosis / Reimplantation / Not defined |
| **Is the vesicouterine pouch obliterated?** | Yes/No |
| **DE posterior to the uterus** | Deep invasive nodule / Adhesions / Both |
| **Location of posterior DE** | Rectovaginal without bowel involvement / Bowel / USL without rectovaginal endometriosis |
| **Rectovaginal DE without bowel involvement** | Up to 2 lesions |
| → Were lesion dimensions recorded? | Yes/No |
| → Dimensions of lesion | X × Y × Z (mm) |
| → Surgical procedure | Excision / None |
| → Was the vagina opened and sutured? | Yes/No |
| **Is the bowel affected by endometriosis?** | Yes/No. If yes: |
| → Number of lesions | 1–3 |
| → Location of lesion | Lower rectum <6 cm / Mid rectum 6–10 cm / Upper rectum >10 cm / Sigmoid colon |
| → Depth of invasion | Serosa / Partial thickness / Full thickness |
| → Involvement of vagina | Yes/No |
| → Involvement of USL | Yes/No |
| → Were lesion dimensions recorded? | Yes/No |
| → Dimensions of lesion | X × Y × Z (mm) |
| → Surgical procedure | Serosal shaving / Partial-thickness excision / Full-thickness excision / Bowel resection / None |
| → Was the vagina opened and sutured? | Yes/No |
| **DE in the USL?** | Right / Left / Torus |
| → Were lesion dimensions recorded? | Yes/No |
| → Dimensions of lesion | X × Y × Z (mm) |
| → Surgical procedure | Excision / None |
| **Is the POD obliterated?** | Yes/No. If yes: |
| → Extent of obliteration | Complete / Retrocervical / To the middle of the posterior uterine wall / Towards the uterine fundus |
| **DE outside the pelvis?** | Yes/No |
| **Was all DE resected?** | Yes/No |
| **If no, why was complete resection not performed?** | Free text |

DE = deep endometriosis, USL = uterosacral ligament

Table S3. Diagnostic performance of transvaginal ultrasound and combined transvaginal ultrasound with magnetic resonance imaging in detecting deep endometriosis and endometriomas with surgical visualization as reference standard. A combined test result was defined as positive when at least one modality was positive and negative only when both modalities were negative, n=107.

|  |  | **Accuracy%** 95%CI | **Sens%**; 95%CI | **Δ Sens%** | **Spec%**; 95%CI | **Δ**  **Spec**  **%** | **PPV%**; 95%CI | **NPV%**; 95%CI | **LR+**; n | **LR-**; n | **TP**  n | **FP**  n | **TN**  n | **FN**  n |
| --- | --- | --- | --- | --- | --- | --- | --- | --- | --- | --- | --- | --- | --- | --- |
| **Endometrioma**  n = 86  Prev 80.4%  95%CI  71.6–87.4 | **TVS** | 94.4  88.0-  97.5 | 96.5  90.1- 99.3 | +2.3  0.3 – 8.1 | 85.7  63.7- 97.0 | -19.0  -41.9 –  -5.4 | 96.5  90.1- 99.3 | 85.7  63.7- 97.0 | 6.76  2.37- 19.28 | 0.04  0.01- 0.13 | 83 | 3 | 18 | 3 |
|  | **TVS+MRI** | 92.5  85.7-  96.2 | 98.8  93.7- 100.0 |  | 66.7  43.0- 85.4 |  | 92.4  84.9- 96.9 | 93.3  68.1- 99.8 | 2.97  1.62- 5.43 | 0.02  0.00- 0.13 | 85 | 7 | 14 | 1 |
| **Urinary tract**  **(Urinary bladder and ureters)** | **TVS** | 82.2  73.7-  88.4 | 42.9  21.8- 66.0 | +4,8  0.1 – 23.8 | 91.9  83.9- 96.7 | -9.3  -17.5 –  -4.1 | 56.2  83.9- 96.7 | 86.8  78.1- 93.0 | 5.27  2.22- 12.50 | 0.62  0.43- 0.91 | Bladder: 1  Ureters: 8  Any: 9 | 2  5  7 | 101  82  79 | 3  12  12 |
| n = 21  Prev 16.6%  95%CI  12.6–28.4 | **TVS+MRI** | 75.7  66.6-  83.0 | 47.6  25.7- 70.2 |  | 82.6  72.9- 89.9 |  | 40.0  21.1- 61.3 | 86.6  77.3- 93.1 | 2.73  1.44- 5.19 | 0.63  0.42- 0.96 | Bladder: 2  Ureters: 9  Any: 10 | 7  8  15 | 96  79  71 | 2  11  11 |
| **Bowel**  n = 58  Prev 54.2%  95%CI  44.3-63.9 | **TVS** | 72.0  62.6-  79.7 | 81.0  68.6- 90.1 | +10.3  3.9 – 21.2 | 61.2  46.2- 74.8 | -6.1  -16.9 – -1.3 | 71.2  58.7- 81.7 | 73.2  57.1- 85.8 | 2.10  1.44- 3.04 | 0.31  0.17- 0.55 | 47 | 19 | 30 | 11 |
|  | **TVS+MRI** | 74.8  65.6-  82.2 | 91.4  81.0- 97.1 |  | 55.1  40.2- 69.3 |  | 70.7  59.0- 80.6 | 84.4  67.2- 94.7 | 2.04  1.48- 2.80 | 0.16  0.07- 0.38 | 53 | 22 | 27 | 5 |
| **USLs**  n = 48  Prev 44.9  95%CI  35.2-54.8 | **TVS** | 58.9  49.2-  67.9 | 58.3  43.2- 72.4 | +29.2  17.0–44.1 | 59.3  45.7- 71.9 | -20.3  -32.8 –  -11.0 | 53.8  39.5- 67.8 | 63.6  49.6- 76.2 | 1.43  0.97- 2.12 | 0.7  0.47- 1.04 | 28 | 24 | 35 | 20 |
|  | **TVS+MRI** | 60.7  51.1-  69.6 | 87.5  74.8- 95.3 |  | 39.0  26.5- 52.6 |  | 53.8  42.2- 65.2 | 79.3  60.3- 92.0 | 1.43 1.14- 1.81 | 0.32 0.14- 0.72 | 42 | 36 | 23 | 6 |
| **Obliterated POD**  n = 62  Prev 57.9  95%CI  48.0-67.4 | **TVS** | 70.1  60.7-  78.1 | 98.4  91.3- 100.0 | +1,6  0.0 – 8.7 | 31.1  18.2- 46.6 | -13.3  -26.8 –  -5.1 | 66.3  55.7- 75.8 | 93.3  68.1- 99.8 | 1.43  1.17- 1.74 | 0.05  0.01- 0.38 | 61 | 31 | 14 | 1 |
|  | **TVS+MRI** | 65.4  55.8-  73.9 | 100  94.2- 100.0 |  | 17.8  8.0- 32.1 |  | 62.6  52.3- 72.1 | 100  63.1- 100.0 | 1.22  1.06- 1.39 | 0.00  .  . | 62 | 37 | 8 | 0 |

n = number, CI = confidence interval, TVS = Transvaginal Ultrasonography, MRI = Magnetic Resonance Imaging, USL = uterosacral ligament, POD = pouch of Douglas, Prev = prevalence, Sens = sensitivity, Spec = specificity, PPV = positive predictive value, NPV = negative predictive value, TP = true positive, TN = true negative, FN = false negative, LR+ = positive likelihood ratio, LR- = negative likelihood ratio, Δ = delta, differences.

Table S4. Diagnostic performance of transvaginal ultrasound for detecting deep endometriosis and endometriomas, using histological diagnosis as the reference standard, n = 189.

|  |  | **Accuracy%**; **95%CI** | **Prev%**; **95%CI** | **Sens%**; **95%CI** | **Spec%**; **95%CI** | **PPV%**; **95%CI** | **NPV%**; **95%CI** | **LR+**; **n** | **LR-**; **n** |
| --- | --- | --- | --- | --- | --- | --- | --- | --- | --- |
| **Endometrioma.**  **histological verification** | **TVS** | 78.5  69.6  -85.3 | 70.1  60.5- 78.6 | 92.0  83.4- 97.0 | 46.9  29.1- 65.3 | 80.2  70.2- 88.0 | 71.4  47.8- 88.7 | 1.73  1.24- 2.41 | 0.17  0.07- 0.40 |
|  | **TVS+MRI** | 78.5  69.6  -85.3 | 70.1  60.5- 78.6 | 96.0  88.8- 99.2 | 37.5  21.1- 56.3 | 78.3  68.4- 86.2 | 80.0  51.9- 95.7 | 1.54  1.17- 2.02 | 0.11  0.03- 0.35 |
| **Bowel,**  **histological verification** | **TVS** | 67.2  57.8-  75.6 | 47.7  37.9- 57.5 | 80.4  66.9- 90.2 | 55.4  41.5- 68.7 | 62.1  49.3- 73.8 | 75.6  59.7- 87.6 | 1.80  1.31- 2.48 | 0.35  0.19- 0.65 |
|  | **TVS+MRI** | 68.2  58.7-  76.4 | 47.7  37.9- 57.5 | 90.2  78.6 96.7 | 48.2  34.7- 62.0 | 61.3  49.4- 72.4 | 84.4  67.2- 94.7 | 1.74  1.33- 2.28 | 0.20  0.08- 0.49 |
| **USL,**  **histological verification** | **TVS** | 63.6  53.9-  72.2 | 38.3  29.1- 48.2 | 65.9  49.4 79.9 | 62.1  49.3- 73.8 | 51.9  37.6- 66.0 | 74.5  61.0- 85.3 | 1.74  1.19- 2.54 | 0.55  0.35- 0.88 |
|  | **TVS+MRI** | 54.2  44.6-  63.5 | 38.3  29.1- 48.2 | 85.4  70.8- 94.4 | 34.8  23.5- 47.6 | 44.9  33.6- 56.6 | 79.3  60.3- 92.0 | 1.31  1.05- 1.63 | 0.42  0.19- 0.94 |
| **Urinary tract,**  **(Urinary bladder and ureters) histological verification** | **TVS** | 87.9  80.1-  92.9 | 10.3  5.2- 17.7 | 63.6  30.8- 89.1 | 90.6  82.9- 95.6 | 43.8  19.8 70.1 | 95.6  89.1- 98.8 | 6.79  3.16- 14.60 | 0.40  0.18- 0.88 |
|  | **TVS+MRI** | 81.3  72.7-  87.7 | 10.3  5.2- 17.7 | 72.7  39.0- 94.0 | 82.3  73.2- 89.3 | 32.0  14.9- 53.5 | 96.3  89.7- 99.2 | 4.11  2.34- 7.21 | 0.33  0.13- 0.87 |

TVS = Transvaginal ultrasonography, MRI = Magnetic Resonance Imagine, USL = uterosacral ligament, POD = pouch of Douglas, Prev = prevalence, Sens = sensitivity, Spec = specificity, PPV = positive predictive value, NPV = negative predictive value, LR+ = positive likelihood ratio, LR- = negative likelihood ratio

Table S5. Case volume and site-specific diagnostic performance of transvaginal ultrasound by individual examiner in the surgical cohort (n=189)

| **Examiner** | **No. of examinations** | **Endometrioma** | | **Urinary tract** | | **Bowel** | | **USL** | | **POD** | |
| --- | --- | --- | --- | --- | --- | --- | --- | --- | --- | --- | --- |
|  |  | **Sensitivity** | **Specificity** | **Sensitivity** | **Specificity** | **Sensitivity** | **Specificity** | **Sensitivity** | **Specificity** | **Sensitivity** | **Specificity** |
| 1 | 23 | 14/14 (100%) | 8/9 (88.9%) | 1/2 (50.0%) | 21/21 (100%) | 6/9 (66.7%) | 12/14 (85.7%) | 1/7 (14.3%) | 15/16 (93.8%) | 7/9 (77.8%) | 8/14 (57.1%) |
| 2 | 18 | 11/12 (91.7%) | 6/6 (100%) | 0/1 (0%) | 16/17 (94.1%) | 6/6 (100%) | 9/12 (75.0%) | 4/6 (66.7%) | 5/12 (41.7%) | 5/5 (100%) | 7/13 (53.8%) |
| 3 | 3 | 2/3 (66.7%) | NE | NE | 3/3 (100%) | 0/2 (0%) | 1/1 (100%) | 1/2 (50.0%) | 1/1 (100%) | 2/2 (100%) | 1/1 (100%) |
| 4 | 4 | 2/2 (100%) | 1/2 (50.0%) | NE | 4/4 (100%) | NE | 3/4 (75.0%) | 1/1 (100%) | 3/3 (100%) | NE | 3/4 (75.0%) |
| 5 | 16 | 4/4 (100%) | 12/12 (100%) | NE | 16/16 (100%) | 1/1 (100%) | 15/15 (100%) | 0/3 (0%) | 13/13 (100%) | NE | 13/16 (81.3%) |
| 6 | 121 | 77/80 (96.3%) | 32/41 (78.0%) | 8/18 (44.4%) | 97/103 (94.2%) | 37/46 (80.4%) | 61/75 (81.3%) | 23/39 (59.0%) | 64/82 (78.0%) | 51/54 (94.4%) | 46/67 (68.7%) |
| 7 | 4 | NE | 4/4 (100%) | NE | 4/4 (100%) | NE | 4/4 (100%) | NE | 4/4 (100%) | NE | 4/4 (100%) |
| **Overall** | **189** | **110/115 (95.7%)** | **63/74**  **(85.1%)** | **9/21**  **(42.9%)** | **161/168 (95.8%)** | **50/64**  **(78.1%)** | **105/125 (84.0%)** | **30/58**  **(51.7%)** | **105/131 (80.2%)** | **65/70**  **(92.9%)** | **82/119**  **(68.9%)** |

Data are presented as n/N, USL = uterosacral ligaments, POD = pouch of Douglas, NE = not estimable. Examiner-specific estimates should be interpreted cautiously because case volumes were highly uneven across sonologists, particularly for less prevalent anatomical sites.
